# Closing the Door on Quantum Nonlocality

**DOI:** 10.3390/e20110877

**Published:** 2018-11-15

**Authors:** Marian Kupczynski

**Affiliations:** Département d’informatique et d’ingénierie, Université du Québec en Outaouais, Case postale 1250, succursale Hull, Gatineau, QC J8X 3X7, Canada; marian.kupczynski@uqo.ca; Tel.: +1-819-777-4493

**Keywords:** Bell-inequalities, quantum nonlocality, computer simulations of Bell tests, local causality, contextuality loophole, photon identification loophole

## Abstract

Bell-type inequalities are proven using oversimplified probabilistic models and/or counterfactual definiteness (CFD). If setting-dependent variables describing measuring instruments are correctly introduced, none of these inequalities may be proven. In spite of this, a belief in a mysterious quantum nonlocality is not fading. Computer simulations of Bell tests allow people to study the different ways in which the experimental data might have been created. They also allow for the generation of various counterfactual experiments’ outcomes, such as repeated or simultaneous measurements performed in different settings on the same “photon-pair”, and so forth. They allow for the reinforcing or relaxing of CFD compliance and/or for studying the impact of various “photon identification procedures”, mimicking those used in real experiments. Data samples consistent with quantum predictions may be generated by using a specific setting-dependent identification procedure. It reflects the active role of instruments during the measurement process. Each of the setting-dependent data samples are consistent with specific setting-dependent probabilistic models which may not be deduced using non-contextual local realistic or stochastic hidden variables. In this paper, we will be discussing the results of these simulations. Since the data samples are generated in a locally causal way, these simulations provide additional strong arguments for closing the door on quantum nonlocality.

## 1. Introduction

In classical physics, we are used to thinking that physical observables characterizing a certain state of a physical system have definite values, even if they are not measured. This assumption is sometimes called counterfactual definiteness (CFD). When we look at a table, we may define its different attributes: its size, dimensions, weight, the type of material it is made of, and so forth. It is natural to think that definite values of these attributes do exist, even if we do not measure them, and that by making more and more precise measurements we may estimate these definite values with arbitrary precision. Even in classical physics, many of these attributes are relative to their experimental context, such as temperature, a place on the Earth, ambient light, and so forth. Nevertheless, we may still believe that the attributes have definite values in each particular fixed environmental context. According to CFD, we may describe ensembles of physical systems using joint probability distributions of several physical variables, even if we do not measure them. CFD was applied with success in statistical mechanics, but failed to explain the motion of electrons in atoms and in many other quantum phenomena.

According to quantum mechanics (QM), as Peres stated, unperformed experiments have no results [1]. In QM, outcomes of measurements are produced and recorded after the interaction of measuring instruments with physical systems in well-defined experimental contexts [2,3]. The measurements of incompatible quantum observables require mutually exclusive experimental set-ups. A joint probability distribution of the outcomes of these measurements does not exist, and probabilistic models using such counterfactual distributions fail to describe the outcomes of these measurements. The measurements of incompatible observables provide only complementary contextual information about studied physical systems. One may say that the classical filters are selectors of pre-existing properties, in contrast to quantum filters which are creators of contextual properties. In mathematical language, the lattices of classical and quantum filters are incompatible [4,5,6,7]. 

QM predicts strong correlations between outcomes of spin polarization measurements in an idealized EPRB experiment, performed on “photon pairs” prepared in a singlet state [8,9]. Several spin polarization correlation experiments (SPCE) have been performed in order to verify and confirm these predictions [10,11,12,13,14,15]. In twin-photon beam experiments, Alice and Bob may choose two settings for their respective polarization beam splitters (PBS) and study correlations between clicks detected on their detectors. Since only one pair of settings may be used at a given time, the results will form four samples coming from four, mutually exclusive, random experiments.

In 1964, John Stewart Bell constructed a local realistic hidden-variable model (LRHVM) in which the clicks on detectors were completely determined by some hidden variables describing each pair of photons prepared in the spin singlet state. LRHVM is CFD-compliant because the outcomes of all spin measurements are predetermined before the actual measurements are done. Using a unique parameter space and a joint probability distribution, to describe four mutually exclusive random experiments, he proved his famous inequalities and showed that according to QM they should be violated for some experimental settings [16,17]. For LRHVM, one does not assume that various spin projections may be measured simultaneously, in all directions, on a given “photon pair”; one only assumes that there exists a probability distribution of some hidden variables from which all probabilistic predictions for feasible pairs of experiments may be deduced. Local realistic hidden-variable models are isomorphic to particular Kolmogorov models using the joint probability distributions of all possible values of spin projections [18,19,20,21]. Similar inequalities were derived using the stochastic hidden variable model (SHVM) in which the experimental outcomes are produced in independent random experiments run at distant locations [17,22].

Idealized EPRB experiments cannot be realized in the laboratory; since we cannot follow “entangled photon pairs” from the moment when they are produced to the moment when they arrive to the detectors. We may only record the clicks on distant detectors and the time of their registration. To compare experimental outcomes with quantum predictions, one has to identify which clicks correspond to the “twin-photon pairs” we are interested in. These experimental uncertainties are known as: time-coincidence, detection, fair-sampling, or photon identification loopholes [23,24,25,26,27,28,29]. The final post-selected data samples in real experiments strongly depend on the photon identification procedures used. Setting-dependent post-selection is also a source of the apparent violation of Einsteinian no-signaling reported in the literature [30].

Various Bell-type inequalities are violated by quantum predictions and by experimental data [10,11,12,13,14,15]. In spite of several claims that all the loopholes have been closed, according to recent experiments, we share the opinion that it is impossible to perform a completely loophole-free Bell experiment [28,29]. However, at the same time, we have no doubt that the violation of various Bell-type inequalities has been confirmed, and that in fact, no more Bell experiments are needed. The violation of inequalities is not surprising. 

It was pointed out by several authors [7,18,19,20,21,28,29,30,31,32,33,34,35,36,37,38,39,40,41,42,43,44,45,46,47,48,49,50,51,52,53,54,55,56,57,58,59,60,61,62,63,64,65,66,67,68,69,70,71,72,73], whereby the list of references is by no means complete, that all these inequalities are proven using CFD and/or oversimplified probabilistic models which are inconsistent with the experimental protocols used in SPCE. A detailed discussion of the intimate relationship between experimental protocols and probabilistic models may be found, for example, in [7,30,63].

Bell believed that LRHVM and SHVM were the only possible local descriptions of EPRB; thus, the violation of his inequalities would mean that QM violates Einsteinian locality. In [3] he gave his first definition of *quantum nonlocality*: “In a theory in which parameters are added to quantum mechanics to determine the results of individual measurements, without changing the statistical predictions, there must be a mechanism whereby the setting of one measurement device can influence the reading of another instrument, however remote. Moreover, the signal involved must propagate instantaneously, so that such a theory could not be Lorentz invariant.” When the violation of Bell and CHSH inequalities was confirmed in SPCE, he concluded that a causally local explanation of quantum correlations was impossible, and that Nature was non-local. 

Let me provide a simple explanation on why and how a possibility of the violation of Einsteinian locality is discussed in the context of an idealized EPRB experiment. Let us assume that Harry prepares “photon pairs” in a spin-singlet state, and sends one photon to Alice and another photon to Bob. After passing by respective PBSs, chosen in a particular pair of settings, the photons produce clicks on one of two detectors placed behind both Alice’s and Bob’s PBSs, respectively. The clicks are interpreted as the detections of photons either ”spinning up” or “spinning down” in a direction determined by the settings of PBSs, and observed events are coded by ±1. 

According to QM, clicks are produced in a perfectly random way, with a 50% chance of getting “+1” and “−1”, respectively. “Twin photons” do not know which setting will be chosen when travelling to Alice and Bob. Now, according to some interpretations of EPRB experiment, for some settings, if Alice registers “+1”, then Bob always registers “−1”, and vice versa. A new law of Nature responsible for the existence of perfect correlations between outcomes of distant random experiments is called *quantum nonlocality*. Two photons are often compared to two fair dice in distant locations, which, in each throw, produce perfectly matching outcomes. By definition of randomness, perfectly random outcomes cannot always match perfectly. This means that some spooky instantaneous influences exist between distant dices, which are forbidden by Einstein’s locality. Since experimental settings may only be defined by small spherical angles, QM does not predict strict anti-correlations between Alice’s and Bob’s outcomes [54,56] even for idealized EPRB experiments. As mentioned above, in real SPCE experiments, the situation is much more complicated and observed correlations may be explained without violating causal locality [30,63].

LRHVM and SHVM failed to explain quantum correlations because they are inconsistent with the contextual character of quantum observables. In LRHVM, twin photons are described as if they were a pair of Bertelsmann’s socks [7,17], characterized by different sizes, matching colors, and tissues. Harry prepares a mixed statistical ensemble of these pairs of socks, sending one sock to Alice and another sock to Bob. Alice and Bob register their properties and code their results by “+1” and “−1”. The outcomes are predetermined, and a measurement process is passive (CFD). A standard joint probability distribution, describing the ensemble of socks prepared by Harry, may be used to deduce all pair-wise correlations of interest. LRHVM is able to describe perfect correlations, but these correlations may not violate Bell-inequalities.

In SPCE, we do not see “pairs of photons” produced by a source. For each pair of settings, we record only “±1” outcomes which correspond to the clicks produced by these invisible photons after their interaction with PBSs and detectors. In contrast to the socks, these outcomes “±1” are not attributes of invisible photons. For each setting the statistical scatter of recorded “±1” can be described by its own specific probabilistic model. For four pairs of settings we have four probabilistic models consistent with predictions of QM, which may not be obtained as marginal probability distributions from some joint probability distributions of quadruples (±1, ±1, ±1, ±1), which does not exist. The existence of such a joint probability distribution is necessary in order to prove Bell-type inequalities. 

In SHVM, “twin photon pairs” are described as a pair of dice. For each pair of settings, Harry prepares setting-dependent pairs of dice, sending one dice to Alice and another to Bob. Alice and Bob throw their dice several times, estimating the probabilities of possible outcomes. This experimental protocol is impossible to implement for pairs of photons, and is inconsistent with the experimental protocol used in SPCE. Moreover, for SHVM, one may always construct a joint probability distribution describing all these experiments.

LRHVM fails because it does not include supplementary-setting dependent variables which describe measuring instruments. As Theo Nieuwenhuizen said, the *contextuality loophole* is fatal for the derivation of Bell inequalities [65,66,67]. If such parameters are correctly incorporated in probabilistic models, quantum correlations may be explained in a locally causal way [30,63].

SHVM fails because if the clicks are produced in a random way, then a memory of the correlations between the photons, created by a source, is destroyed. Some nontrivial correlations may also exist when the mixed-ensemble of pairs of dice is used by Harry, but they may never violate Bell-type inequalities [60]. 

A notion of *quantum nonlocality* is often associated with the notion of perfect *nonlocal randomness*. Randomness is a subtle notion, which is discussed in detail, for example, in [42,52]. When we flip a fair coin, we say that heads or tails appear at random with a probability of 50%. However, if we knew what the initial conditions were, we could, in principle, predict the outcome. This is called imperfect classical randomness. It is often believed that a true randomness is a quantum intrinsic randomness according to which quantum phenomena can produce perfectly random events that may not be explained using classical randomness. 

In statistics, we deal with many random experiments. To verify whether outcomes appear randomly, one has to perform several randomness tests. Please note, however, that the digits which appear in the decimal development of the number π pass, with success, all the tests of randomness, while at the same time being strictly determined. Einstein believed that quantum probabilities were not intrinsic and that they should emerge from some underlying, more detailed theory of natural phenomena. The failure of SHVM to explain quantum correlations may be perhaps indication that he was right. A more detailed discussion of LRHVM and SHVM may be found in [7].

LRHVM and SHVM may be simulated in computer experiments. For several years, Hans de Raedt, Kristel Michielsen, and collaborators [74,75,76,77,78,79,80,81] have simulated several quantum experiments, event-by-event and in a locally causal way, including EPRB. In this paper, we focus our discussion on recent papers [29,80,81]. We explain the probabilistic meaning of their algorithms and their results in a simple and concise way.

The authors, using algorithms consistent with LRHV or with SHVM, simulate outcomes of real experiments. Estimated correlations using raw data are not consistent with quantum predictions for EPRB. The agreement with QM is obtained using specific setting-dependent photon identification procedures, mimicking those applied in real experiments, and are used to extract final experimental samples. In contrast to real experiments, the authors may also generate computer outputs for counterfactual experiments; such as instantaneous measurements performed in all the different settings, for successive correlated “photon pairs”. The authors prove that the correlations between distant clicks, estimated using the raw data from these counterfactual computer experiments, satisfy exactly all the Bell-type inequalities for any finite sample. 

It does not matter whether the outcomes are predetermined or not. What matters is the existence of some joint probability distribution of counterfactual outcomes (where each equal “±1”).

This provides explicit proof that Bell-type inequalities hold only if the correlations may be deduced from the data drawn from a statistical population described by a joint probability distribution of all possible outcomes. Such a joint probability distribution does not exist for SPCE. Many years ago, George Boole derived similar inequalities. Therefore, Bell inequalities should perhaps be called Boole-Bell inequalities [46,50,70,71]. 

Since computer simulations are explicitly causally local, they close the door on speculations about mysterious quantum nonlocality, which is believed to be responsible for the correlations between distant outcomes studied in SPCE.

This paper is organized as follows:

In Section 2, we discuss the results of the papers [80,81], in which outcomes are generated using an algorithm consistent with LRHVM, and “photon pairs” are identified using setting-dependent registration times.

In Section 3, we discuss the results of the paper [29], in which outcomes are generated using an algorithm consistent with SHVM and “photons“ are locally identified using voltage traces, compared with some detection thresholds.

In Section 4, we discuss the effect of post-selections on the properties of extracted samples.

In Section 5, we explain what we mean by saying that QM is a contextual theory, and we clarify the definition of the *contextuality loophole*.

In Section 6, we discuss how computer simulations may contribute to testing whether QM theory is *predictably complete* (able to explain all properties of experimental data).

Section 7 contains several conclusions.

## 2. Computer Simulation Experiments Using Registration Time Delays

As we mentioned in the introduction, according to CFD, a “photon pair” in SPCE is described before a measurement by predetermined values of spin projections in all directions In CFD-compliant LRHVM, joint probability distribution on a unique-probability space is used to deduce the correlations between spin projections for different pairs of settings used in SPCE.

In their computer experiments [80,81], Hans de Raedt, Kristel Michielsen, and Karl Hess generated, for each member of a “photon pair”, measurement outcomes and time delays of their registration. At first, time delays were not taken into consideration. All outputted outcomes were used to estimate the correlations between clicks in “distant laboratories” and to verify the validity of CHSH inequality [82,83].Subsequently, using outputted time delays and their differences, correlated “photon pairs” were identified, as in real experiments [11], and post-selected data samples were consistent with the predictions of QM for EPRB.

The authors used the following CFD-compliant algorithm. A “photon pair” entering observation stations was represented by (φ, φ + π/2, r1, r2). A polarizer was described by (*a*, *T*), where *T* is a fixed parameter related to a time unit and *a* is an angle of a chosen setting. The two lines below explain how an outcome *x* and *a* time delay t* are calculated, for each “photon” passing by a polarizer:

compute c = cos [2(*a* − φ)]; s = sin [2(*a* − φ)] (1)

set *x* = sign(c); t* = *r·T·s*^2^ (2)

where φ is randomly drawn from [0, 2π], and *r* from [0, 1]. Please note that outcomes *x* do not depend on the parameters *r*; thus, these parameters could be assigned not to the incoming “photon pairs” but to the observation stations. Such an interpretation is adopted in the paper [29], which we discuss in the next section.

Two different protocols are used to generate data samples. We call them Protocol 1 (implementable) and Protocol 2 (counterfactual).

- Protocol 1 is consistent with a realizable protocol in SPCE. Outcomes for any pair of settings are generated, at each time, for a different “photon pair”. Thus, four pseudo-random time series of data for four different pairs of settings are generated:
  (3) $$(x_{1}(t),x_{2}(t),t_{1}^{*}(t),t_{2}^{*}(t))=G(\varphi_{1}(t),\varphi_{2}(t),r_{1}(t),r_{2}(t),a_{1}(t),a_{2}(t))\;$$
  where *t* = 1, …, 4N and (a_1_(*t*), a_2_(*t*)) = (a_1_, a_2_) for *t* = 1, …, N; (a_1_(*t*), a_2_(*t*)) = (a_1_, a’_2_) for *t* = N + 1, …, 2N; (a_1_(*t*), a_2_(*t*)) = (a’_1_, a_2_) for *t* = 2N + 1, …, 3N; (a_1_(*t*), a_2_(*t*)) = (a’_1,_ a’_2_) for *t* = 3N + 1, …, 4N.
- Protocol 2 is impossible to implement in SPCE and is forbidden by QM. For each “photon pair” predetermined outcomes and time delays, for all available settings, are outputted. A generated pseudo-random time series is:
  (4) $$(x_{1}(t),x^{\prime }{}_{1}(t),x_{2}(t),x^{\prime }{}_{2}(t),t_{1}^{*}(t),t^{\prime }{}_{1}^{*}(t),t_{2}^{*}(t),t^{\prime }{}_{2}^{*}(t))=G_{1}(\varphi_{1}(t),\varphi_{2}(t),r_{1}(t),r_{2}(t),a_{1},a^{\prime }{}_{1},a_{2},a^{\prime }{}_{2})\;$$
  where *t* = 1, …, 4N.

The authors used a different definition of CFD [29,80,81]. According to their definition, Protocol 1 is CFD non-compliant and Protocol 2 is CFD-compliant. According to our definition, we call a protocol CFD–compliant if, and only if, it generates outcomes which are predetermined by the parameters describing a “photon-pair”.

In [84], Richard Gill studied the impact of CFD-compliance on samples which were created in an idealized SPCE experiment with random choices of settings. He defined a 4 × 4N counterfactual spreadsheet. Each line of this spreadsheet describes each incoming “photon pair” by predetermined values “±1” of spin projections for four settings (a, a’, b, b’) used in SPCE. If no constraints are imposed, one can have only 16 different lines in the spreadsheet which are permuted randomly, depending on a sequence of “photon pairs” arriving to detectors.

In SPCE, pairs of settings are chosen randomly and only the results for one pair of settings can be observed for each incoming pair. Gill constructs experimental samples by choosing, from each line of the spreadsheet, two outcomes corresponding to randomly chosen settings. Finite samples, created following this protocol, may not violate CHSH inequality as significantly as it was predicted by QM, and Gill makes a conjecture:

(5) $$\;\mathrm{Pr}\left({\langle AB\rangle }_{obs}+{\langle AB^{\prime }\rangle }_{obs}+{\langle A^{\prime }B\rangle }_{obs}-{\langle A^{\prime }B^{\prime }\rangle }_{obs}\geq 2\right)\leq \frac{1}{2}\;$$

where <*AB*>*_obs_*, <*AB’*>*_obs_*, <*A’B*>*_obs_*, and<*A’B’*>*_obs_* are the correlations for the different pairs of settings estimated using the counterfactual spreadsheet 4 × 4N, if N tends to infinity. 

Samples containing outcomes *x*_1_, *x*’_1_, *x*_2_, and *x*’_2_, created using Equation (1)–(4), have the same properties as the samples studied by Gill. Namely:

- Protocol 1 chooses, for each “photon pair“, only two entries from a corresponding line of the spreadsheet—one for Alice, and one for Bob. It is obvious from (3) that the first N pairs of entries are chosen from the same pair of columns since the settings are changed systematically, only for at *t* = N + 1, 2N + 1, 3N + 1, 4N + 1. In spite of the fact that pairs of settings are not chosen randomly, generated samples have the same properties as the samples in [84].
- Protocol 2 chooses, for each “photon pair”, a complete line of the counterfactual spreadsheet. These lines form a sample drawn from some CFD-compliant joint probability distribution of four random variables. It is easy to see from [62,84] that in this case, |S| = 2 for any finite sample—thus, CHSH inequality is never violated.

For Protocol 1, results of computer simulations before a post-selection are consistent with Gill’s conjecture (54 samples out of 100 violate |S| ≤ 2). For Protocol 2, CHSH inequality is never violated, and it also confirms the result of simulations of Sasha Vongher [85]. Vongher demonstrated that if a fate of a “photon” is predetermined before the measurement, one may not, under the assumption of a fair sampling, violate the Bell and CHSH inequalities as significantly as it is predicted by QM [62,85]. All computer simulations confirm that LRHVM is not consistent with QM.

In order to identify which pairs of computer outputs correspond to “photon pairs”, the authors post-select their final data samples by comparing the differences between time delays with suitable time windows W. Two outcomes are identified as a photon pair, if $|t_{2}^{*}(t)-t_{1}^{*}(t)|\leq W$. Correlations estimated using these post-selected setting-dependent samples agree remarkably well with the correlations predicted by QM. Moreover, the time-window dependence of these estimates reproduces such dependence observed in a real experiment [11]. 

## 3. Computer Simulation Experiments Using Detection Thresholds

In [29], the authors constructed a faithful model of the laboratory experiments of Giustina et al. [14] and Schalm et al. [15], performed to check Eberhard [86] and Clauser–Horn inequalities [22]. Clauser–Horne inequalities are derived using SHVM. In contrast with LRHVM, the outcomes are not predetermined by the variables describing the inputted “photon pair”. In SHVM:

(6) $$P(x_{1},x_{2}|a_{1},a_{2})=\sum_{\lambda \in \Lambda }P(\lambda_{1},\lambda_{2})P(x_{1}|a_{1},\lambda_{2})P(x_{2}|a_{2},\lambda_{2})\;$$

where (*λ*_1_, *λ*_2_) describes “photon pairs”; (a_1_, a_2_) are chosen settings; and *P*(*x*_1_|a_1_, *λ*_1_) and *P*(*x*_2_|a_2_, *λ*_2_) are probabilities describing stochastically independent measurements, performed in distant laboratories by Alice and Bob. If (*λ*_1_, *λ*_2_) are continuous variables, the sum in (6) is replaced by an integral. A more detailed discussion of SHVM may be found in [7,17,22].

In [29], the following (SHVM-compliant) algorithm is used:

- For each *k* = 1, …, N, a uniform random generator generates two floating-point numbers $0\leq \phi_{1,k}\leq 2\pi$ and $\phi_{2,k}=\phi_{1,k}+\pi /2$, which are inputted to the stations with settings (a_1_, a_1_’) and (a_2_, a_2_’), respectively. The *k*-th event simulates the emission of a “photon pair” with maximally correlated, orthogonal polarizations for all the essential features of the laboratory experiments.
- Upon receiving the input, an observation station generates two pseudo-random numbers $\left(r,\hat{r}\right)$ and computes:
  (7) c=cos[2(a−ϕ)], s=sin[2(a−ϕ)] 
  and sets:
  (8) $$x=sign\left(1+c-2\cdot r\right),\;v=\hat{r}|s{|}^{d}\left(Vmax-Vmin\right)-Vmax\;$$
  where *d* is an adjustable parameter, and positive numbers *Vmin* and *Vmax* define the range of the voltage traces produced on the detectors by the incoming signals. Voltage signal *v* is identified as a “single photon” event if, and only if, −*Vmax* ≤ *v* ≤ *V* ≤ −*V min,* where *V* is a chosen detector threshold.

The choice of the specific functional forms of *x* and *ν* in (7) and (8) is inspired by a similar model which employs time-coincidence to identify pairs and exactly reproduces the single particle averages and two-particle correlations of the singlet state, if the number of events becomes very large [75,79].

The Equation (7), for each value of φ and uniformly distributed values of *r,* generates a sequence of randomly distributed *x*, such that the empirical frequency distribution of *x* = 1 and *x* = −1 agrees with Malus’ law:

(9) $$P(X=1|a,\varphi )=\cos^{2}(a-\varphi )\;and\;P(X=-1|a,\varphi )=\sin^{2}(a-\varphi )\;$$

where *X* is a random variable taking values *x*.

Voltage traces are only used to identify correlated “photon pairs”, so we discuss, for the moment, the properties of generated samples of outcomes. Using the Equations (7) and (8), the authors perform two computer experiments following two different protocols. Now the outcomes *x* are not predetermined, and we call them Protocol 1 and Protocol 2.

- Protocol 1 (implementable) is consistent with the experimental protocol used in SPCE because the outcomes, for any pair of settings, are generated each time for a different “photon pair”. Namely, four pseudo-random time series of data for four different pairs of settings may be generated:
  (10) $$(x_{1}(t),x_{2}(t))=F_{1}(\varphi_{1}(t),\varphi_{2}(t),r_{1}(t),r_{2}(t),a_{1}(t),a_{2}(t))\;$$
  where *t* = 1, …, 4N and (a_1_(*t*), a_2_(*t*)) = (a_1_, a_2_) for *t* = 1, …, N; (a_1_(*t*), a_2_(*t*)) = (a_1_, a’_2_) for *t* = N + 1, …, 2N; (a_1_(*t*), a_2_(*t*)) = (a’_1_, a_2_) for *t* = 2N + 1, …, 3N; (a_1_(*t*), a_2_(*t*)) = (a’_1,_ a’_2_) for *t* = 3N + 1, …, 4N. The fact that the settings are not randomly chosen has no impact on the properties of generated samples.
- Protocol 2 (counterfactual) is impossible to realize in SPCE and QM, because it calculates and outputs, at each step, four pseudo-random values (simulating the outcomes of a simultaneous joint measurement of incompatible observables performed on each “photon pair”). The generated pseudo-random time series of outputs for the four simultaneous measurement settings may be defined as:
  (11) $$(x_{1}(t),x^{\prime }{}_{1}(t),x_{2}(t),x^{\prime }{}_{2}(t))=F_{2}(\varphi_{1}(t),\varphi_{2}(t),r_{1}(t),r_{2}(t),{r^{\prime }}_{1}(t),{r^{\prime }}_{2}(t)a_{1},a^{\prime }{}_{1},a_{2},{a^{\prime }}_{2})\;$$
  where *t* = 1, …, 4N.

Samples produced using Protocol 1 are consistent with a specific SHVM model (6):

(12) $$P(x_{1},x_{2}|a_{1},a_{2})=\int_{0}^{2\pi }d\varphi_{1}\int_{0}^{2\pi }d\varphi_{2}P(\varphi_{1},\varphi_{2})P(x_{1}|a_{1},\varphi_{1})P(x_{2}|a_{2},\varphi_{2})\;$$

where the probability density $P(\varphi_{1},\varphi_{2})=\delta (\varphi_{2}-\varphi_{1}-\pi /2)/{\left(2\pi \right)}^{2}$ and the probabilities $P(x_{1}|a_{1},\varphi_{1})P(x_{2}|a_{2},\varphi_{2})$ are calculated using the Malus law (9). Using (9), we can easily find:

(13) $$E(X_{1})=E(X_{2})=0\;\mathrm{and}\;E(X_{1}X_{2})=\frac{1}{2}\;\cos2(a_{1}-a_{2})\;$$

where *X*_1_ and *X*_2_ are the random variables which take the values *x*_1_ = ±1 and *x*_2_ = ±1, respectively.

Here, the single expectation values vanish, as predicted by QM. QM predicts for EPRB similar expectation values $E(X_{1}X_{2})=\;\cos2(a_{1}-a_{2})$. However, the expectation values $E(X_{1}X_{2})$, displayed in (13) contain a factor ½, meaning that they do not violate CHSH inequality. The agreement with quantum predictions is obtained only after the “photon identification procedure”, which selects, from the raw data, final data samples. 

In contrast with Protocol 1, any finite sample of quadruples $\left(x_{1}(t),x^{\prime }{}_{1}(t),x_{2}(t),x^{\prime }{}_{2}(t)\right)$ generated using protocol 2 trivially obeys all Bell and CHSH inequalities, similarly to the computer simulations discussed in the preceding section. Again, the data may be displayed using a 4 × 4N counterfactual spreadsheet, and it does not matter that now its entries are not predetermined.

This confirms that Bell-inequalities are only a necessary and sufficient condition for the existence of a joint probability distribution for three dichotomous random variables *X*_i_ taking the values *x_i_* = ±1, which can be measured pairwise, but not all simultaneously. As mentioned in the introduction, the similar inequalities were already derived by Georges Boole [87] and generalized by Vorob’ev [88]. This is why these inequalities are violated in successive spin polarization measurements [89], in some experiments in social sciences [90,91,92], and even in classical mechanics [59]. Claims that their violations imply mysterious *quantum nonlocality* or *super-determinism* are unfounded [63].

As we can see, the raw data of computer simulations are not consistent with some quantum predictions. To produce data samples consistent with QM, the authors have to apply a setting-dependent “photon-pair identification procedure”, mimicking the procedures used in real experiments. In the next section, we discuss the influence of post-selection on the properties of the selected samples. 

## 4. Setting–Dependent Post-Selection

It is well-known that, in order to obtain reliable information about some statistical population, one has to study *simple random* samples drawn from this population. A random sample is simple if all trials are independent and identically distributed.

In computer simulations compliant with LRHVM or with SHVM, *simple pseudo-random* samples can be generated. These samples are not consistent with quantum probabilistic predictions, and specific setting-dependent post selection of data items is needed. Similarly, raw data from real experiments may not be compared with theoretical predictions without applying “photon pair” identification procedures. These procedures contain free parameters, such as time windows, registration thresholds, and so forth. The properties of the post-selected samples vary significantly in the function of these parameters [11,26,30]. It is well-known that models which incorporate setting-dependent post selection and/or exploit so-called coincidence loopholes may produce results [23,24,25] which violate CHSH inequality (|S| ≤ 2).

To illustrate the possible dramatic impact of post-selection, let us consider two perfect simple random samples, S_1_ = {*x*_1_, …, *x*_n_} and S_2_ = {*y*_1_, … ,*y*_n_}, where *x*_i_ = ±1 and *y*_i_ = ±1. Using a simple pairing of outcomes, we obtain a sample S_3_ = {(*x*_1_, *y*_1_), …, (*x*_n_, *y*_n_)}, for which *E*(*XY*) = 0.

- If we post-select data items, using a criterion “kept only if *x*_i_ + *y*_i_ = 2”, we extract a completely correlated sub-sample of S_3_, for which *E*(*XY*) = 1.
- If we post-select data items, using a criterion “kept only if *x*_i_ + *y*_i_ = 0”, we extract a completely anti-correlated sub-sample of S_3_, for which *E*(*XY*) = −1.
- If we randomly post-select data items from S_3_, we obtain an uncorrelated sub-sample for which (*E*(*XY*) = 0).

In spite of the fact that the post-selection in SPCE is necessary and justified, we share the opinion that a loophole-free test of Bell-type inequalities may not be performed [28,51]. We demonstrated with Hans de Raedt [93] that sample inhomogeneity invalidates standard significance tests. Despite the fact that sample homogeneity could not be or was not tested carefully enough in Bell experiments [10,11,12,13,14,15], we do not doubt that Bell-type inequities have been violated in these experiments. In view of our discussion presented in the preceding sections, the violation of Bell-type inequalities is by no means surprising, and new tests are simply not needed. 

It is sometimes claimed that the setting-dependence of experimental samples may only be explained by some mysterious influences between distant experimental set-ups, super-determinism, or conspiracy of detectors whose efficiencies change in order to comply with QM. However, there is a much simpler explanation—the setting-dependence of experimental samples, in real experiments and in computer ones, reflects the active role played by measuring instruments. The measurement outcomes are not predetermined, and depend on the experimental settings.

Quantum observables are contextual, and for each of the settings used in SPCE, the data may only be described by a specific setting-dependent probabilistic model. Probabilistic proofs of Bell-type inequalities suffer from the *contextuality loophole* because they try to describe various incompatible experiments using setting-independent hidden variables [66,67].

In the next section, we explain in more detail what we mean by the *contextuality loophole.* We also demonstrate how the setting-dependent photon identification procedures produce data samples consistent with specific probabilistic models which do not suffer from this loophole.

## 5. The Meaning of the Contextuality Loophole

*Contextuality* has a different meaning for different authors [7,44,49,54,57,66,67,94,95,96,97,98]. For some authors, a theory is non-contextual if values of physical observables do not depend on a specific experimental protocol used to measure these observables. For example, a length of a table does not depend on how the length was measured. Similarly, QM does not say how the linear momentum of an electron and its energy are measured. The quantum state vector represents all equivalent preparation procedures, and a self-adjoint operator represents all equivalent measurement procedures of a corresponding physical observable [98]. Therefore, one can find statements in the literature that QM, along with classical physics, is a non-contextual theory [96].

Physical systems may have different properties. Some of them are attributive, which means that they do not change in their interaction with measuring instruments, and they do not depend on the environment in which they are measured. Attributive properties are, for example, the rest mass and the electric charge of an electron. Physical systems may be also described by contextual properties, which are only revealed in particular experimental and/or environmental contexts, such as color, weight, magnetization, spin projection, and so forth.

An interesting contextual property is a probability (understood not as a subjective belief of a human agent). A probability is neither a property of a coin nor a property of a flipping device; it is only a property of a whole random experiment [7,21,42,49,57].

In quantum theory, as Bohr insisted, we deal with “the impossibility of any sharp distinction between the behavior of atomic objects and the interaction with the measuring instruments which serve to define the conditions under which the phenomena appear” [2]. In particular, QM gives probabilistic predictions on a statistical scatter of outcomes obtained in repeated “measurements” of some physical observable on “identically prepared physical systems”. Since the probability is only a property of a random experiment performed in a particular experimental context, QM provides a contextual description of the physical reality.

Similarly, Kolmogorov’s probabilistic models provide a contextual description of random experiments. Namely, each random experiment (in which there is a statistical stabilization) is described by its Kolmogorov model defined on a dedicated probability space [7,42,52,63]. Probabilistic models, as well as QM, do not enter into details on how individual outcomes are obtained, but provide only predictions for the experiments as a whole. If the context of an experiment changes (for example, where we have two slits open instead of one), then the Kolmogorov and quantum probabilistic models change. This is why we say that QM is a contextual theory. Only rarely different random experiments may be described using marginal probabilities deduced from some joint probability distributions [7,48,52,63], and the joint probability distributions of incompatible quantum observables do not exist.

Following Theo Nieuwenhuizen [66,67], we say that the proofs of Bell-type inequalities suffer from the fatal *contextuality loophole*. We also say that a probabilistic hidden variable model suffers from the *contextuality loophole,* if it uses the same probability space to describe different incompatible random experiments. To close the *contextuality loophole*, a model has to incorporate supplementary variables which describe measuring devices [65,66,67]. If setting-dependent parameters are correctly incorporated in a hidden-variable probabilistic model, then Bell-type inequalities may not be proven. If *x*_a_(t) = f_a_(*λ*_1_(t), *λ*_a_(t), a) and *x*_b_(t) = f_b_(*λ*_2_(t), *λ*_b_(t), b), where (*λ*_1_(t), *λ*_2_(t)) and (*λ*_a_(t), *λ*_b_(t)) describe an “EPR pair” and the “microstates” of measuring instruments in the setting (a, b), Gill’s counterfactual spreadsheet does not exist and the only constraint we have is |S| ≤ 4. The same constraint was derived by Andrei Khrennikov in his generalization of the Kolmogorov model for SPCE experiments [99,100]. A detailed discussion of a model which is able to reproduce any correlations in SPCE may be found in [63].

Therefore, any hidden-variable model wanting to reproduce quantum predictions must include explicit dependence on the settings. In [29,80,81], “photon identification procedures” allow for the generation of samples which may be described by contextual, setting-dependent probabilistic models, able to exactly reproduce quantum predictions even for idealized EPRB experiments. For example, equation 19 from [76], rewritten in an explicitly contextual form, is as follows:

(14) $$\;P\left(x_{1},x_{2}|\alpha_{1},\beta_{1},W\right)=\overset{2\pi }{\underset{0}{\int }}\overset{2\pi }{\underset{0}{\int }}P\left(x_{1}|\alpha ,\xi_{1}\right)P\left(2|\beta ,\xi_{2}\right)P\left(\xi_{1},\xi_{2}|\alpha ,\beta ,W\right)d\xi_{1}d\xi_{2}\;$$

where *W* is chosen time-window, (*α*_1_ = *α*, *β*_1_ = *β*) are chosen settings, and (*ξ*_1_, *ξ*_2_) are some local setting-dependent hidden variables. The probability distribution of (*ξ*_1_, *ξ*_2_) depends on (*α*, *β*, *W*). The setting dependence in these models is a result of locally causal data generation, and any speculations about a spooky action in the distance are unfounded Contextual probabilistic models of SPCE may also be defined in a more direct and intuitive way, such as in [62,63]. 

To describe the time-series of data in detail in various domains of science, we usually have to use stochastic processes. It is reasonable to ask whether quantum probabilities may explain all fine structures in experimental time-series [53,101,102,103]. Computer simulations, which enter into details on how individual data items are created, allow for the modelling of real experiments and for incorporating time delays and time-windows, which is impossible in QM. Successful computer simulations of several quantum experiments demonstrate how quantum probabilities might emerge from a more detailed description of quantum phenomena. Testing whether QM is *predictably complete* (able to explain all properties of experimental data) is necessary and it does not depend on the existence of such detailed description. This is the topic of the next section. 

## 6. Computer Simulations and Predictable Completeness of QT

Bohr claimed that any subquantum analysis of quantum phenomena is impossible, and that QM provides a complete description of individual physical systems [99]. Einstein believed that quantum probabilities were emergent and that a complete theory should give more details on how individual outcomes are created [104,105]. Bohmian mechanics, stochastic electrodynamics, hidden-variable models, and various computer simulations are attempts to realize this program in particular cases. Several quantum phenomena have been successfully simulated [74,75,76,77,78,79,80,81]. Event-by-event simulations of quantum experiments are trying to get more intuitive understandings of quantum phenomena. These simulations have no ambition of replacing QM. They generate time series of outcomes, similar to those created in real experiments, and study effects which QM is unable to address. Different simulation protocols, based on the same probabilistic model, provide more detailed information on how data are created and may produce significantly different finite samples [93]. 

In real experiments [11,12,13,14,15], one uses time-windows and time delays in order to identify correlated detection events. The main ingredient of the computer model, as discussed in section 2, is also time delays. The authors say that these time delays are due to the existence of dynamic many-body interactions of a photon with a measuring apparatus. In our opinion, the Equations (1) and (2) allow for us to imagine more detailed mechanisms on how data in the real experiment might have been created.

Let us change slightly the assignment of the variables used in (1) and (2), and describe an “EPR-pair” by (φ, φ + π/2) and measuring devices by (*a*_1_, r_1_, T; *a_2_*, r_2_, T). Before entering a PBS, the ”magnetic moments” of each pair are pointing in opposite directions. During the interaction with the PBSs, they are “aligned” along the directions a_1_ and a_2_, respectively, and are subsequently “sent” to corresponding detectors. It is plausible that the time needed for this alignment increases and decreases in the function of (*a* − φ). The model would have been closer to this physical intuition with parameters *r* drawn randomly from [1 − *c*, 1], where *c* is a small positive number. We see from (2) that if r is close to 0, the outputted time delay is negligible, no matter which setting is chosen.

Of course, the dependence of time delays on *(a* − φ) in (2) was chosen to reproduce quantum predictions. The possibility of the existence and implications of time delays in Bell experiments were discussed in detail for the first time by Saverio Pascazio [24]. The dependence of time delays on the settings was searched but not found in an EPRB experiment with random-variable analyzers [106]. .In our opinion it would be interesting to search for such time delays in dedicated experiments. with linearly polarized beams of photons. If such time delays were discovered, it would give an additional argument in favor of the idea that QM is not *predictably complete*. 

In several papers, we advocated that in order to check the *predictable completeness* of QM, one has to search for unexpected regularities in the experimental time-series of data [53,101,102,103]. Some interesting regularities in time-series from Bell experiments have already been discovered by using nonlinear analyses of data [107,108].

## 7. Conclusions

In classical physics, statistical ensembles of physical systems may often be described by probabilistic models using joint probability distributions of several physical variables, even if we do not measure all of them at the same time. It is assumed that a measurement of any particular property does not change other properties which are not measured. 

LRHVM describes only a counter-factual random experiment in which, for each “photon-pair”, all spin-projections are simultaneously measured by Alice and Bob in their available experimental settings. If outcomes are coded “±1”, the recorded data form quadruples (±1, ±1, ±1, ±1) and pair-wise correlations estimated using these data may never violate Bell inequalities. Pair-wise correlations for different pairs of settings in Bell-experiments are estimated using the data coming from mutually exclusive random experiments, and they do not need to satisfy the constraints resulting from the existence of the counterfactual joint probability distribution. 

Apparently, theoretical arguments based on probability spaces and the properties of random variables have not been sufficiently convincing. The outcomes of computer simulations are perhaps easier to understand. Computer simulations allow for the generation of data sets for several counterfactual experiments, such as repeated measurements on the same “photon pair”, joint or successive measurements performed on the same” photon-pair” in different settings, and so forth. 

Using counterfactual protocols, quadruples (±1, ±1, ±1, ±1) are generated for each “photon-pair”. These quadruples may be displayed in a 4 × 4N spreadsheet. If pair-wise correlations are estimated using this spreadsheet, Bell and CHSH inequalities are satisfied for any finite sample. It does not matter whether the outputs ±1 are predetermined [81] or generated randomly [29]; what does matter is that we use quadruples to estimate pair-wise correlations. Using the language of mathematical statistics, we say that quadruples form a finite sample drawn from a statistical population described by some joint probability distributions of four random variables.

Joint probability distributions are only well-defined if we measure several random variables at the same time, such as the weight and height of a student, the numbers on two dice rolled together, and so forth [63]. If we want to study the correlations between outcomes of distant experiments, we must define how the outcomes are paired. The properties of the generalized joint probability distributions (GJPD) describing these paired outcomes depend on how the pairing was defined. A detailed discussion of GJPDs and of pairing protocols may be found, for example, in [7,90,91].

If, in computer simulations, only pairs of outcomes are outputted for each “photon pair”, some finite samples may violate Bell-type inequalities, but not as significantly as in real experiments. Final data samples are created using “photon-pair identification” procedures, mimicking the procedures used in real experiments. Similarly, as in real experiments, post-selected samples depend strongly on time delays, time windows, and voltage thresholds. Each of these post-selected samples is consistent with some specific setting-dependent probabilistic models. For some specific values of adjustable parameters, such models reproduce exact quantum predictions for EPRB experiments. 

Computer simulations provide direct proof that Boole- and Bell-type inequalities are a necessary and sufficient precondition for the existence of GJPD, from which the pair-wise correlations between random variables may be deduced. Such GJPD does not exist for the random variables measured in Bell tests. Moreover, using locally causal algorithms, they give a more detailed description of how individual data items might have been created in real experiments. Such a detailed description is out of the scope of QM and was believed to be impossible. Therefore, we cannot close the door on the Bohr-Einstein quantum debate [63,81], in spite of what was claimed by Aspect [109].

Bell-type inequalities have been violated in [11,12,13,14,15], but it does not matter whether there are spooky influences or not between distant experimental set-ups. It does not matter whether experimentalists have a “free will” to choose their setting or not. The only thing that matters is the impossibility to deduce correlations between distant clicks in SPCE for different pairs of settings, using the same counterfactual GJPD. 

A completely different argument in favor of locality and *contextuality* has recently been given by Kurt Jung [110]. He derived polarization correlations of “photon pairs” in triplet and singlet configurations, using the fact that circularly polarized wave packets associated with entangled “photon pairs” are phase-shifted at the source. “The linear polarization component of a circularly polarized photon is not defined before the measurement has taken place. Nonetheless, the polarization correlation of entangled photons measured at distant locations is well-defined and agrees with the predictions of quantum mechanics.” 

The correlations between distant events in the natural and social sciences have recently been studied by means of integrable systems and nonlinear dynamical modelling. A lot of papers have been published in this new domain called AB science, in which such correlations are explained in a rational way. The discussion of AB science is out of the scope of this paper, and the references may be found in [111].

In EPRB, one is interested in the coincidences between the events in distant locations. It is interesting to mention that, perhaps due to the general relativity, the possibility of exact and absolute coincidence events might be an illusion [112].

Relativistic invariance is incorporated in quantum electrodynamics and in the Standard Model. As with any physical law, it might be violated in high-energy physics, and has to be tested [113,114]. Even if such a violation had been found, it would have not given arguments in favor of *quantum nonlocality* as it is understood in the context of Bell tests. 

We conclude that various theoretical arguments, reviewed in this paper and locally causal simulations of Bell experiments, allow for a “closing of the door” on *quantum nonlocality*. 
